# Allergic Rhinitis in Rats Is Associated with an Inflammatory Response of the Hippocampus

**DOI:** 10.1155/2018/8750464

**Published:** 2018-04-16

**Authors:** Shasha Yang, Jing Wu, Qinxiu Zhang, Xinrong Li, Daien Liu, Bin Zeng, Zhiqing Liu, Haoran Kang, Zhendong Zhong

**Affiliations:** ^1^Chengdu University of Traditional Chinese Medicine, Chengdu, Sichuan province, China; ^2^Guiyang College of Traditional Chinese Medicine, Guiyang, Guizhou province, China; ^3^Department of Otorhinolaryngology, Head and Neck Surgery, Teaching Hospital of Chengdu University of Traditional Chinese Medicine, Chengdu, Sichuan province, China; ^4^Sichuan Academy of Medical Sciences and Sichuan Provincial People's Hospital, Chengdu, China

## Abstract

Allergic rhinitis (AR) is a major concern in personal and public health, which negatively affects emotions and behavior, leading to cognitive deficits, memory decline, poor school performance, anxiety, and depression. Several cellular and molecular mediators are released in the inflammatory process of AR and activate common neuroimmune mechanisms, involving emotionally relevant circuits and the induction of anxiety. Responsiveness of the hypothalamic-pituitary-adrenal (HPA) axis to allergic processes have been reported, which may also include responsiveness of the hippocampus, cortex, and other brain regions. Here, we have used an optimized rat model of AR to explore whether the disease has a relationship with inflammatory responses in the hippocampus. AR was established in adult rats by ovalbumin sensitization, and the expression of various inflammatory substances in the hippocampus was measured by specific assays. Comparison between experimental and various control groups of animals revealed an association of AR with significant upregulation of substance P, microglia surface antigen (CD11b), glial fibrillary acid protein (GFAP), tumor necrosis factor-*α* (TNF-*α*), and interleukin 6 (IL-6) in the hippocampus. Thus, we hypothesize that the AR challenge may activate these inflammatory mediators in the hippocampus, which in turn contribute to the abnormal behavior and neurological deficits associated with AR.

## 1. Introduction

Allergic rhinitis (AR) is an IgE antibody-mediated immune reaction, in which expansion of the type 2 (TH2) subset of T cells [1, 2] causes typical allergy symptoms, consisting of sneezing, itching, nasal congestion, watery rhinorrhea, and even impaired quality of life (QOL) [3–5], affecting sleep, psychomotor functions, and social activities. AR is a highly prevalent chronic disease that affects approximately 40% of the world's population [6]. Consequently, the overall socioeconomic burden and the medical expenses associated with AR have become areas of major concern.

In addition, multiple clinical practitioners have reported behavioral complications associated with the progression of AR, which include cognitive deficits, memory decline, poor school performance, attention deficiency and hyperactivity, anxiety, and depression [7–9], mainly in childhood and adolescent populations. Recent human studies have hinted at a direct relationship between antigen exposure and alteration in brain function that may precipitate high levels of anxiety and emotional reactivity [10, 11]. Several rodent studies have also shown that cellular and molecular mediators are released in the inflammatory process of AR and activate common neuroimmune mechanisms that involve emotionally relevant circuits and the induction of anxiety [12, 13]. Increased anxiety in the open field test and activation of limbic brain regions was reported during the early phase in a mice model of food allergies [14]. In humans, increased brain activity in the prefrontal cortex was observed using functional magnetic resonance during the late phase of an asthma episode [12]. Other hypotheses for the association between allergic diseases and internalizing disorder invoke the role of interleukin 1*β* in hypersensitivity reactions, which activates the hypothalamic-pituitary-adrenal (HPA) axis, stimulating the release of cortisol that in turn modifies serotonin release, leading to mood disturbances [15]. Moreover, mouse models have proposed a direct relationship between antigen exposure and altered brain function leading to increased anxiety. Production of T helper 2 cytokines in the prefrontal cortex and olfactory bulbs of rats with tree pollen- and ovalbumin-induced AR has also been demonstrated [12].

While these studies provide a foundation in identifying brain regions and behavioral responses associated with allergen challenge, the notion that these allergies may initiate, perpetuate, and exacerbate pathological anxiety remains controversial and largely unstudied. Besides, the relationship between allergic rhinitis and anxiety has been shown to be bidirectional and likely to share genetic and neuroimmune mechanisms that remain undefined. Studies of brain regions and behavioral responses associated with AR mostly focused on the limbic brain and prefrontal cortex, much less on the hippocampus, even though the hippocampus is closely related to learning, memory, and emotion, which appear to be affected in AR [16].

The brain and the immune system interact with each other through neurons [17, 18] and body fluids, and the central nervous system (CNS) regulates the HPA axis that includes the hypothalamus, pituitary gland, and adrenal gland. The HPA axis is in fact an important part of the neuroendocrine system, involved in controlling stress and regulating many physical activities, such as digestion, immune system, mood, emotions, and sexuality, as well as energy storage and consumption. In turn, the immune system transmits signals to the brain through neural and humoral routes. Immune organs are innervated by the sympathetic nervous system, and immune cells express receptors for neurotransmitters, including catecholamines and neuropeptides, and for hormones, including those of the HPA axis.

The nasal mucosa is also innervated by sensory, sympathetic, and parasympathetic nerves. Thus, immune activity in the nasal mucosa may be passed into the brain by the body fluid and neural pathways, such as the afferent of cytokines and the vagus nerve. Sensory neurons transmit signals that generate sensations such as itching and motor reflexes such as sneezing, whereas parasympathetic and sympathetic reflexes regulate the glandular and vascular systems [19]. Moreover, due to inflammation or airborne allergens, the integrity of the nasal epithelium is disrupted, and the exposed sensory nerve endings promptly release substance P (SP), calcitonin gene-related peptide (CGRP), and vasoactive intestinal peptide (VIP). Produced in the cell body of C-fibre neurons, these peptides can be transported in granule structures within the cytoplasm to nerve terminals in the CNS. This leads to “central sensitization,” a phenomenon associated with the activation of nociceptive C-fibres [19].

Finally, the glial cells, widely distributed in the CNS, support the blood-brain barrier (BBB) and nourish the neurons; however, their immune function has received increasing attention in recent years, focusing on the microglia (detected as OX42 antibody-reactive) and glial fibrillary acid protein (GFAP) levels. Microglia, in particular, is considered one of the most important immune defenses of the CNS and is also a major source of proinflammatory cytokines in oxidative stress, such as tumor necrosis factor (TNF), nitric oxide, interleukin, and neurotoxic substances. In these conditions, massive proliferation of glial cells results in increased GFAP expression [20]. Additionally, a large number of studies have demonstrated a close association of glial cells with learning, memory, and other cognitive functions [21, 22]. In parallel, a growing body of evidence has implicated a role for cytokines in the normal, nonpathological brain and, hence, in the associated learning and memory behaviors [23]. TNF-*α* is also important for activity-dependent synaptic scaling within the hippocampus [24]. TNF-*α*, as well as multiple interleukins (e.g., IL-6, IL-1, and IL-10) and prostaglandins, can markedly influence cognitive function, primarily memory (reviewed in [25]). High-frequency stimulation in the hippocampus also increases IL-6 mRNA expression [26]. Finally, psychological stress has been associated with increased proinflammatory cytokines such as IL-6 and TNF-*α* in human and animal studies [27].

Together, the brain, the neuroendocrine, and the immune systems appear to be inextricably linked. Based on the accumulated evidence, we hypothesized that AR may activate inflammatory responses of the hippocampus that signals through emotionally relevant circuits and causes anxiety, leading to the abnormal behavior patterns. In the current study, we have used a Sprague-Dawley rat model, optimized in our laboratory, to interrogate whether hypersensitivity reactions and altered behavior in AR are associated with increased glial proliferation and induction of inflammatory response in the hippocampus.

## 2. Materials and Methods

### 2.1. Antibodies and Reagents

The OVA antigen was purchased from Sigma (A8040, USA) and aluminum hydroxide as adjuvant was from Kelong chemical factory (lot number 201110328, Chengdu, Sichuan, China). Enzyme-linked immunosorbent assay (ELISA) kits, specific for IgE (sIgE), interleukin 4 (IL-4), interferon-*γ* (IFN-*γ*), TNF-*α*, and IL-6 were purchased from Abcam (England). Antibodies against SP, GFAP, and CD11b (OX42) and rabbit anti-mouse secondary antibody were also purchased from Abcam, England. Where mentioned, biotin-conjugated rat anti-mouse antibodies were purchased from BD Pharmingen, Beijing, China. Sodium citrate buffer (0.01 M, pH 6.0) was prepared for dilution, where needed. The microscopic image acquisition and analysis system were, respectively, from BA200 Digital and Image-Pro Plus 6.0 (Media Cybernetics, USA).

### 2.2. Animals

Adult, male Sprague-Dawley rats (250–300 g) were obtained from Da Shuo Biological Technology Co. Ltd. (Chengdu, Sichuan, China). Before the experiment began, all rats were adapted for 1 week in the Experimental Animal Center of Chengdu University of TCM. The committee recognized that the proposed experimental procedures complied with the Animal Protection Law. All rats were housed in a temperature-controlled room (22–24°C) under a 12-hour light/dark cycle (7 am–7 pm). Food and water were available ad libitum in home cages.

### 2.3. Establishment of the AR Model

The AR model was established using an ovalbumin (OVA) sensitization method [14]. Rats were sensitized (days 1–13) with 7 intraperitoneal (i.p.) injections of 0.3 mg OVA (Sigma A8040, USA) as antigen and 30 mg aluminum hydroxide as adjuvant dissolved in 1 ml of saline. Following the i.p. immunization, the nasal antigen challenge (days 14–21) was performed with intranasal dripping of 50 *μ*l of 2% OVA daily for 7 consecutive days. The animals in the control group were administrated with the same volume of saline. All animals were closely observed for development of any nasal responses of sneezing and watery rhinorrhea, and scraping, for 30 min after each challenge. Symptoms and signs of AR were then provoked (days 22–24) with intranasal dripping of 80 *μ*l of 1% OVA daily for 3 consecutive days. Ten minutes after the last instillation of 1% OVA, all animals were subjected to i.p. injection of 1% sodium pentobarbital (50 mg/kg) to collect tail venous blood. Serum levels of cytokines sIgE, IL-4, and IFN-*γ* were measured by ELISA to evaluate whether AR was successfully established. The sensitization was maintained with intranasal dripping of 50 *μ*l of 1% OVA every other day until the cytokine results confirmed AR. Finally, blood samples and hippocampus tissue were collected for detection of various other parameters by the corresponding methods.

### 2.4. Assay for Animal Behavior

The number of sneezes, degree of runny nose, and nasal rubbing movements during the 30 min period after the final allergen challenge were recorded in each experimental group. Following superimposition of the recording results, a total score of greater than 5 (>5) was used as the benchmark for successful establishment of AR [24]. In addition, we observed the patterns of emotion-related behavior as external manifestations of the AR, including fur color, physical energy, activity intensity, and food intake.

### 2.5. Specimen Collection

Animals of all groups (*n* = 8) were sacrificed with i.p. injection of 3% sodium pentobarbital (30 mg/kg) and then transcardially perfused with 350 ml 0.9% saline and fixed in solution containing 2% paraformaldehyde and 1.25% glutaraldehyde phosphate buffer solution (pH = 7.2). Blood collected from the femoral artery was subjected to the measurement of sera sIgE, IL-4, and INF-*γ*. The nasal mucosa tissues and the hippocampus tissues were quickly removed and separately postfixed in 4% paraformaldehyde for 72 h at 4°C. They were cut in coronal 4-5 *μ*m sections and slide-mounted for microscopic examination.

### 2.6. Enzyme-Linked Immunosorbent Assay (ELISA)

The sIgE, IL-4 and IFN-*γ* levels in the sera and the TNF-*α* and IL-6 levels in hippocampus homogenates were measured by solid-phase ELISA in accordance with the manufacturer's instructions. Bound immunoglobulin isotypes were detected with biotin-conjugated secondary antibody.

### 2.7. Hematoxylin-Eosin (HE) Staining Analysis

Paraffin sections of nasal mucosa tissue were stained with the hematoxylin and eosin method to examine pathological morphology. These sections were immersed in 4% paraformaldehyde for 4 h and transferred to 70% ethanol. Individual lobes of nasal mucosa biopsy material were placed in processing cassettes, dehydrated through a serial alcohol gradient, and embedded in paraffin wax blocks. Before immunostaining, 5 *μ*m thick nasal mucosa tissue sections were dewaxed in xylene, rehydrated through decreasing concentrations of ethanol, and washed in PBS and then stained with hematoxylin and eosin (H&E). After staining, sections were dehydrated through increasing concentrations of ethanol and xylene. The stained sections were observed in mounting media for image acquisition. The pathological morphology were detected by microscopy at 400x magnification.

### 2.8. Immunohistochemical (IHC) Analysis

Paraffin sections of hippocampus tissue were stained with the streptavidin peroxidase method to examine SP, GFAP, and CD11b expressions. These sections were incubated in 3% hydrogen peroxide (H_2_O_2_)/methanol for 15 min. After washing three times in PBS (pH 7.2–7.4) for 5 min each, the sections were immersed in 0.01 M citrate buffer (pH 6.0) for 5 min and then washed twice with PBS. Nonspecific binding was blocked by incubation with normal goat serum for 20 min at 37°C. These sections were then incubated with rabbit anti-SP, anti-GFAP, and anti-OX42 antibodies (1 : 200 dilution) overnight at 4°C and then with biotinylated goat anti-rabbit IgG for 30 min. Following incubation with the horseradish peroxidase-labeled streptomycin ovalbumin reagent, sections were colored using a concentrated DAB kit. The stained sections were observed in mounting media for image acquisition. The immunopositive cells were detected by microscopy at 400x magnification, and the GFAP- and OX42-positive cells were quantified by the average number of positively stained cells per field.

### 2.9. Statistical Analysis

Data are presented as mean ± standard deviation (SD) (SPSS statistical analysis software, version 20.0). All variables indicated approximately normal distribution and parametric testing and were simultaneously subjected to one-way analysis of variance (ANOVA), followed by post hoc analysis using the Student–Newman–Keuls- (SNK-) *q* test. Differences were considered to be statistically significant at a *P* value < 0.05 (*P* < 0.05).

## 3. Results

**Figure 1:**
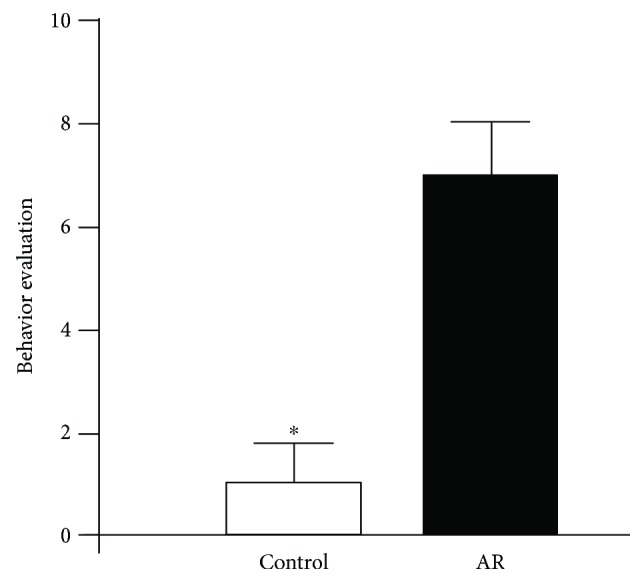
Behavior evaluation between the AR model group and the control group of animals. The total scores in AR model group were greater than 5 (>5), which suggested successful establishment of AR. Data are expressed as mean ± SD; ^∗^*P* < 0.01.

**Figure 2:**
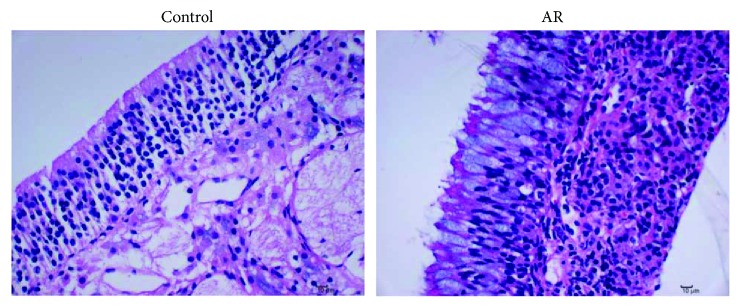


In order to determine whether the AR model was established in the rat, we resorted to three major kinds of parameter points, namely, AR behavior; objective indicators including serum sIgE, IL-4, and IFN-*γ* levels; and HE staining. Our measurement of the behavior score satisfies the AR criteria [28]. Moreover, post hoc analysis revealed that AR behavior scores significantly increased in the model group of rats, compared to the control group (*P* < 0.01, Figure 1). Serum sIgE, in particular, is considered a touchstone diagnostic indicator of AR. Additionally, the levels of IL-4 and IFN-*γ*, respectively, reflect the Th1 and Th2 (T-helper 1and 2) cell populations. Our post hoc analysis revealed that both sIgE and IL-4 significantly increased compared to the control group (*P* < 0.01, Table 1); on the other hand, the levels of IFN-*γ* significantly decreased in the model group [*F*_(1, 14)_ = 12.94, *P* < 0.01]. Nasal mucosa tissues were examined by means of routine HE staining and light microscopy. Results showed that in the AR group of rats, the structural integrity of nasal mucosa tissue was impaired and the ciliated epithelium was irregular with disordered arrangement, exhibiting various degrees of fall-off and edema, and mixed with eosinophils and lymph cells (Figure 2).

Apart from these molecular and cellular indicators of AR, we also observed the emotion-related behavior of the AR and control groups, mostly in external manifestations that specifically included fur color, physical energy, activity intensity, and food intake. Before the sensitization, all rats were normal in daily activities and food intake. However, after the sensitization, the AR group showed external negative manifestations, such as the fur color turned pale or dim, physical energy turned into weakness, activity intensity decreased so that the animals became quiet, and food intake reduced. Overall, the whole body condition was worse than the control group.

**Figure 3:**
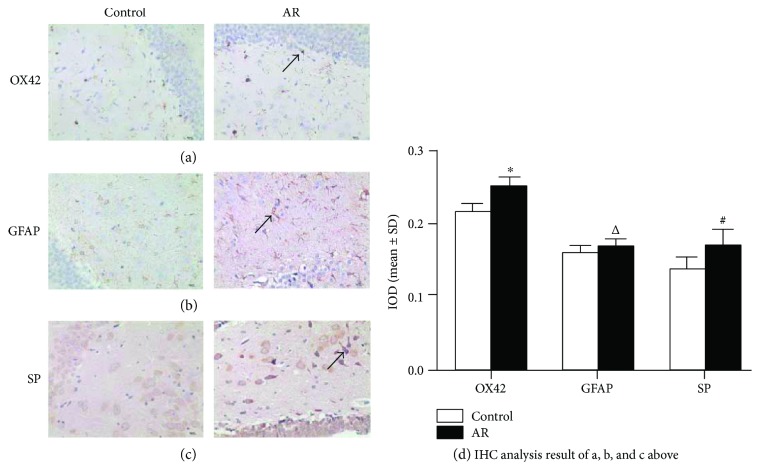
Representative photographs indicating IHC analysis (×400). Expression of (a) CD11b (OX42-stained), (b) GFAP, and (c) SP. (d) shows statistical analysis of the results. Data are expressed as mean ± SD; ^∗^*P* < 0.01, ^△^*P* < 0.05, ^#^*P* < 0.01; *n* = 8; scale bar = 50 *μ*m).

**Figure 4:**
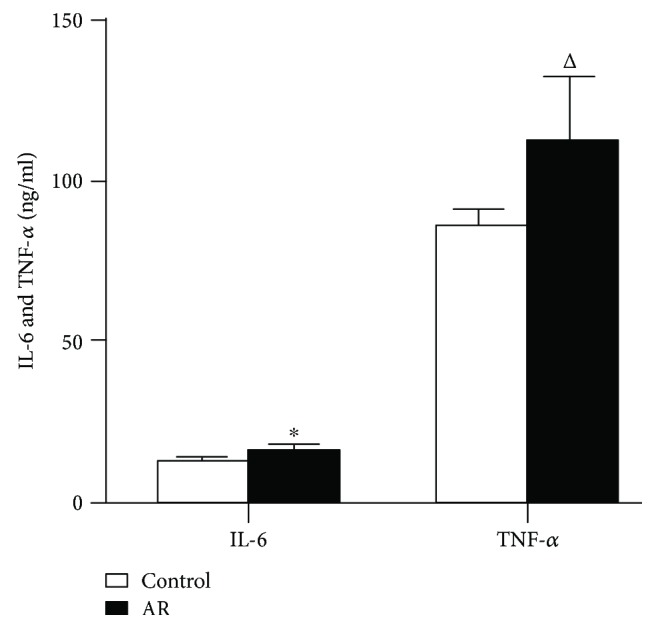
Levels of IL-6 and TNF-a, determined by ELISA. Comparing between in the AR model group and the control group. Data are expressed as mean ± SD; ^∗^*P* < 0.01, ^△^*P* < 0.05; *n* = 8.

In the immunohistochemical (IHC) analysis, we wanted to determine whether inflammation in the hippocampus relates to AR. As shown (Figure 3), the expression of microglial CD11b markers (determined by OX42 antibody staining) and SP in the AR group showed significant increase, compared to the control group (*P* < 0.01, Figures 3(a) and 3(c)). GFAP expression was also significantly higher in the AR group than in the control group [*F*_(1, 14)_ = 5.46, *P* < 0.05, Figure 3(b)]. We also observed that the expression of IL-6 in the AR group was higher than in the control group (*P* < 0.01, Figure 4), as determined by ELISA. Finally, TNF-*α* expression was higher in the AR group compared to the control group (*P* < 0.05; Figure 4). Taken together, the above results show elevated expression of GFAP, SP, IL-6, and TNF-*α*, as well as a higher number of CD11b^+^ glial cells under AR conditions. In particular, SP and IL-6 and CD11+ cells showed significantly higher increase compared with GFAP and TNF-*α* in the AR group. These results clearly indicate that the inflammatory response of the hippocampus correlates with the observed increase of SP and IL-6 and of glial cell activation, which may be affected by nasal hypersensitivity and stimulation. We would like to mention that all rats survived the experiments with no death, and therefore, the observed effects are unlikely due to a general response of severe systemic pathology.

## 4. Discussion

The results presented here document an inflammatory response of the hippocampus in AR. Simultaneously, we observed higher levels of several proinflammatory mediators, which may relate to behavioral changes. Thus, we postulate that in an AR condition, the activated inflammatory response of the hippocampus may involve emotionally relevant circuits, which would then translate into abnormal behavior and negative emotions, mentioned earlier.

To our knowledge, this is the first study to examine the inflammation mechanisms of hippocampus activity in nasal hypersensitivity of a live animal AR model. In this model, we demonstrated upregulation of a significant cohort of proinflammatory molecular markers that included both cytokines as well as glial surface antigen, namely, GFAP, SP, IL-6, TNF-*α*, and CD11b. The OX42-reactive CD11b surface marker is characteristic of brain-specific immune cells, specifically the microglia, and thus, its increased expression in AR rats confirms an immune response. In fact, along with SP and IL-6, the CD11b^+^ microglia population exhibited the most significant increase in the AR group compared to the control group. Moreover, there is a related report that allergy led to a reduced microglia presence and activity and to an elevated level of neurogenesis, such as those of DCX(+) cells and BrdU(+) cells in the hippocampus of allergic mice [29].

Nevertheless, previous studies reported that other brain regions such as the amygdala and hypothalamus are also responsive to respiratory allergies as measured by the expression of c-fos [30, 31]. In addition, functional imaging studies strongly implicated the prefrontal cortex (PFC) in providing inhibitory functions for anxiety and depression [32]. Another study reported Th2 cytokine expression in the brain in response to a peripheral immune challenge [12]. On the other hand, mRNA levels of IL-2 were reported to decrease in the PFC under increased anxiety in the elevated plus maze (EPM) test [33]. As indicated previously, no studies have yet addressed the relationship between behavioral change resulting from AR hypersensitivity and hippocampus activity in a defined laboratory animal model.

While the expression of TNF-*α*, IL-6, GFAP, and SP, together with microglial activation, follows the pattern of a Th2 cell-dependent allergic reaction, the exact mechanism of their increase or function in the hippocampus is unknown at this time. It is tempting to speculate that increases in these cytokines may provide the link between the hippocampus and behavioral changes. The recognition that AR is a clinical condition, capable of influencing behavioral responses, has far-reaching significance in preventing AR development. If AR is exacerbated or not controlled properly, it may cause brain cognitive and memory dysfunctions or other adverse behaviors such as attention deficit and mental disorders, to name a few. Thus, our study suggests that it is important to reduce the occurrence of AR and manage patients with AR for pathological as well as social and economic reasons.

Although this study provides important new information about the inflammatory response of the hippocampus in the AR process, it has some limitations. First, we did not evaluate emotion-related behavior in view of a lack of objective scores but rather used external manifestations in the AR rats, such as fur color, physical energy, activity, and food intake, none of which may accurately reflect emotion-related behavior. Second, the exact mechanisms underlying the observed hippocampus inflammatory response in the AR rats are likely to be more complex, and possibly other neuroimmune mediators involved in emotional changes remain to be elucidated. In the future, more elaborate and sophisticated experiments need to be considered and designed, such as the use of 24-hour video surveillance and objective assessment methods to monitor AR behavior, in order to further investigate the relationship between the hippocampus and AR. Objective behavioral assessments may include emotional behavior evaluations, including anxiety scores and depression scores. In clinical experiments, we can use multiple detection methods such as functional magnetic resonance imaging (fMRI) and positron emission tomography (PET) to investigate the hippocampus of AR patient-related behavior, for instance, the potential depression- and anxiety-related disorders, memory loss, and cognitive impairment. Nevertheless, our findings open the door to novel insights into the inflammatory response of the hippocampus to allergic rhinitis in the rat model. Additional studies will certainly elucidate the complex neuroimmune relationship between the hippocampus and diverse emotional reactivity disorders. Lastly, our findings emphasize the importance of molecular behavioral studies for comprehensive awareness and prevention of AR. The impact of mental health disorders on society is substantial, especially in children with multiple allergic diseases, and implementing primary prevention activities may be warranted.

## Figures and Tables

**Table 1:** Serum levels of sIgE, IFN-*γ*, and IL-4.

Group	sIgE (ng/ml)	IFN-*γ* (mg/ml)	IL-4 (pg/ml)
Control	72.65 ± 9.17	0.59 ± 0.06	10.74 ± 3.05
AR	124.92 ± 15.56^∗^	0.29 ± 0.07^△^	16.04 ± 3.81^#^

Sera were collected from the control group and the AR model group of rats, and the levels of the indicated molecules were measured as described in Materials and methods. Data are expressed as mean ± SD (^∗^*P* < 0.01, ^△^*P* < 0.01, ^#^*P* < 0.01).
